# Pulmonary Manifestations in Rheumatological Diseases

**DOI:** 10.7759/cureus.29628

**Published:** 2022-09-26

**Authors:** Gaurang M Aurangabadkar, Milind Y Aurangabadkar, Sumer S Choudhary, Saood N Ali, Shafee M Khan, Ulhas S Jadhav

**Affiliations:** 1 Respiratory Medicine, Datta Meghe Medical College, Datta Meghe Institute of Medical Sciences, Nagpur, IND; 2 Rheumatology, Arthritis and Rheumatism Clinic, Nagpur, IND; 3 Respiratory Medicine, NKP Salve Institute of Medical Sciences, Nagpur, IND; 4 Respiratory Medicine, Jawaharlal Nehru Medical College, Datta Meghe Institute of Medical Sciences, Wardha, IND

**Keywords:** pulmonary arterial hypertension, bronchiectasis, interstitial lung disease, limited systemic sclerosis, rheumatoid arthriitis, dermatomyositis, systemic lupus erythematosus

## Abstract

Pulmonary involvement complicates the various aspects of care in patients suffering from autoimmune disorders. The epidemiological data generated over the last 10 to 15 years have improved the overall understanding of the risk factors and pathophysiological mechanisms involved in pulmonary involvement in rheumatological conditions. Recent advances in genetics have provided superior insight into the pathogenesis of autoimmune diseases and the underlying pulmonary involvement. This review article provides a concise overview of the four most common rheumatological conditions associated with pulmonary involvement: systemic lupus erythematosus (SLE), dermatomyositis/polymyositis, rheumatoid arthritis (RA), and systemic sclerosis (SSc). The clinical, epidemiological, and genetic aspects of these diseases are summarized in this article with particular emphasis on the characteristic patterns of pulmonary involvement in radiological imaging and various treatment options for each of these autoimmune diseases and their lung manifestations.

## Introduction and background

In rheumatological conditions, auto-immune mediated pulmonary damage is a relatively frequent finding. Pulmonary involvement in these patients is a major factor that influences the disease prognosis, affecting both morbidity and mortality. There is a considerable variation in the modes of lung presentation in patients affected by rheumatic diseases, with some diseases having characteristic presentation patterns concerning the lung structures involved (Table 1) [1].

**Table 1:** Presentation patterns of pulmonary involvement in various rheumatic diseases. (The +/- signs denote the relative prevalence of each pattern of presentation, with – being none, + being low, and +++ being high) PAH: pulmonary arterial hypertension ILD: Interstitial lung disease Cited and modified from  [1]

RHEUMATIC DISEASE	PAH	PLEURAL	AIRWAYS	ILD
Systemic lupus erythematosus	+	+++	+	+
Myositis	+	-	-	+++
Systemic sclerosis	+++	-	-	+++
Rheumatoid arthritis	+	++	+ +	++

In systemic lupus erythematosus (SLE) and rheumatoid arthritis (RA), there is a broad spectrum of pulmonary involvement affecting almost all respiratory tract structures, including both upper and lower respiratory tracts. In contrast, myositis and systemic sclerosis (SSc) patients show predominantly interstitial lung disease (ILD). The earliest clues could be lung manifestations in predicting or diagnosing the risk of developing the rheumatic disease in the future. Depending on the underlying rheumatic disease, the specific cytokines and immune cells could be different even though they share a common pathophysiological mechanism of auto-immune mediated pulmonary injury.

The most typical manifestation of rheumatic disease-associated pulmonary involvement has been found to be ILD, with usual interstitial pneumonia (UIP) being the most frequently encountered pattern [2]. The rheumatic-disease-associated ILDs (RD-ILD) are classified as per the European Respiratory Society (ERS)/American Thoracic Society (ATS) classification revised in 2013, which categorized the various idiopathic interstitial cases of pneumonia (IIPs).

Temporal and spatial heterogeneity alongside areas of the normal lung parenchyma, fibroblastic foci, inflammation of the interstitium, and honeycombing, are the histopathological hallmarks of UIP.

On high-resolution computed tomography (HRCT), the characteristic findings are peripheral reticular opacities with a basal lung predominance, traction bronchiectasis, honeycombing, and minimal or absent ground-glass opacities (GGO) [3]. The non-specific interstitial pneumonia (NSIP) pattern, in contrast to UIP, shows a fairly homogenous appearance, primarily due to inflammatory mononuclear cell infiltration along with interstitial fibrosis of differing magnitudes. The HRCT findings in the NSIP pattern are GGOs and reticular shadowing with relatively preserved lung parenchymal architecture and no honeycombing [4]. The most common types of ILD in rheumatological diseases are UIP and NSIP [5,6].

It has been demonstrated in a study by Joo et al. that UIP associated with rheumatic disease has an overall better prognosis when compared to idiopathic pulmonary fibrosis (IPF) or idiopathic UIP [7]. It would be worthwhile to look for clinical evidence of rheumatological diseases in newly diagnosed cases of IPF. The objective of this review article is to update clinicians regarding pulmonary involvement in rheumatic diseases, namely: SLE, RA, myositis, and SSc, with special reference to its epidemiology, clinical features, pathogenesis, and therapeutic options.

## Review

Rheumatoid arthritis-associated pulmonary involvement

Almost all components of the pulmonary structure are potential targets for injury in patients with RA with lung involvement [1]. In more than 50% of unsegregated RA patients, HRCT studies revealed bronchiectasis and ILD as the most common pulmonary abnormalities, followed by involvement of the pleura and the pulmonary vasculature [8,9]. The majority of pulmonary diseases are first encountered in the first five years after the diagnosis of RA [10], with the earliest manifestation being airway disease [11]. ILD is associated with a poor prognosis for the patient and needs early and specific attention [12]. In the general population, the lifetime risk of ILD has been found to be 0.9%, as compared to 7.7% in patients with RA [12]. More than 50% of RA-ILD patients have UIP as the predominant histopathological pattern, as compared to other rheumatic diseases in which NSIP is by far the most frequently found pattern [6].

Most studies [13,14] identify the following risk factors for RA-ILD as given in Table 2:

**Table 2:** Risk factors associated with an increased risk of developing interstitial lung disease in patients with rheumatoid arthritis RA: Rheumatoid arthritis [13]

RISK FACTORS FOR RA-ILD
Male sex
Elderly patients
History of tobacco smoking
Increased titers of rheumatoid factor
Elevated titers of anti-cyclic citrullinated protein antibodies [Anti- CCP antibodies]
RA severity
Duration of RA

RA-associated ILD may vary clinically from relatively symptom-free to a rapid worsening of disease status. The rate of clinically significant disease progression varies according to the decline in lung function, disease extent, and, most importantly, the histopathological pattern of the ILD. The factors associated with an overall dismal prognosis were found to be a rapidly progressive decline in lung function, extensive disease, and a UIP pattern on histopathology [13,15]. The death hazard ratio in RA-ILD cases was found to be tripled as compared to those patients without the same [12]. Post-ILD diagnosis, the five-year mortality rate in patients can range from 35% to 39% [12,16].

Rheumatic-disease-associated ILD has been found to be rarely associated with an acute exacerbation (AE), which may prove to be fatal and is associated with sudden respiratory status deterioration [17], and is characterized in radiological studies by the onset of the development of new consolidations or GGOs with underlying superimposed reticular shadowing. This condition has been found to be linked to 2.5-fold increased mortality risk in subjects with RA-ILD, with about 64% of the patients dying during the initial episode of acute exacerbation [18]. The factors associated with AE in patients with RA were found to be a history of methotrexate therapy, UIP pattern, and older age at the time of diagnosis of ILD. The clinical course for non-UIP RA-associated ILD has been found to be different as compared to the UIP pattern in HRCT studies [19]. The UIP pattern was linked to a rapid decline in lung function, an increased frequency of hospitalization, and the requirement of oxygen therapy during the same [20]. The elevated mortality rate in RA-ILD patients can be partly explained by the higher prevalence of the UIP pattern.

Genetic Mechanisms Involved in RA and RA-Associated ILD

**Figure 1:**
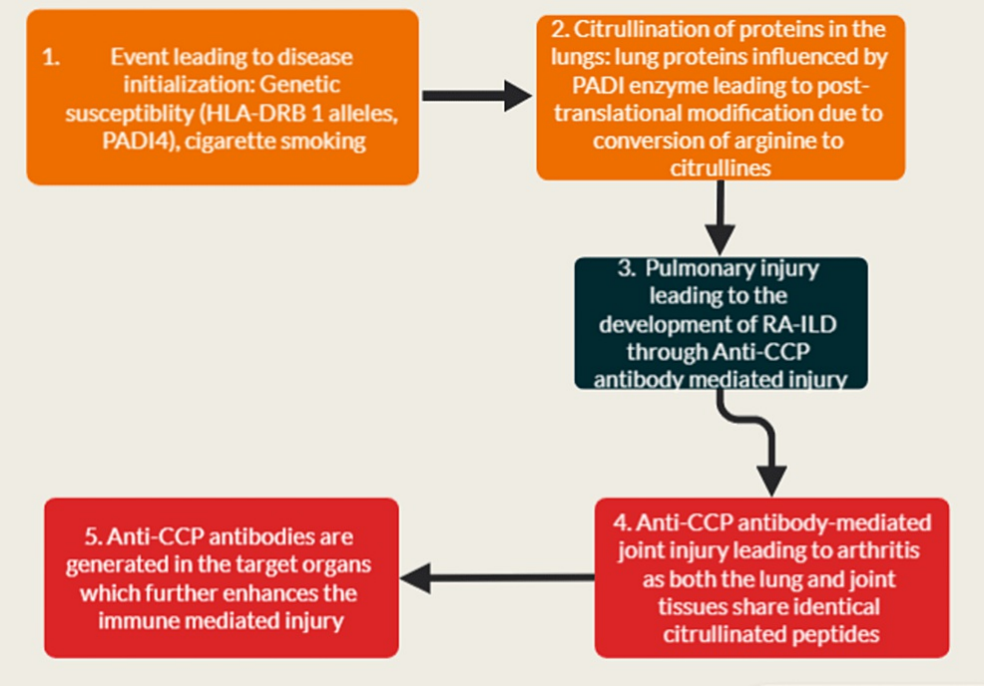
A schematic diagram showing the common pathogenetic pathways involved in the development of both RA and RA-ILD PADI4: Peptidyl arginine deiminase 4 HLA DRB1: Human leukocyte antigen major histocompatibility complex, class II, DR Beta 1 RA-ILD: Rheumatoid arthritis-associated interstitial lung disease Anti-CCP: Anti-cyclic citrullinated peptide Image credits: Original flowchart made by Dr. Gaurang Aurangabadkar

The two genetic factors that are most closely implicated in the development of RA were found to be major histocompatibility complex, class II, DR Beta 1 (HLA-DRB1) alleles, and shared epitope (SE). These alleles were demonstrated to have an elevated affinity for binding to citrullinated proteins in contrast to normal proteins [21]. The shared pathogenesis, especially concerning the immunological and genetic mechanisms of RA and RA-associated ILD, has been depicted in Figure 1.

Tobacco smoking acts as a trigger for the citrullination of protein in the pulmonary tissues [22]. The development of anti-CCP antibody-positive RA can be attributed to the interaction between SE and smoking [23]. For both RA and RA-ILD, smoking has been recognized to be an important risk factor [14], as well as sharing two disease predictors in common, namely: anti-CCP antibodies and RA Factor. An inference from these findings can be that, particularly in smokers, RA starts in the lungs and that the associated pulmonary involvement is a sequela of the environmental and genetic interaction as shown in Figure 1. Therefore, smoking can be considered as a variable that adds insult to the injury, leading to the triggering of anti-CCP antibody production in the lung tissues (Figure 1). Table 3 summarizes the findings from various studies [24-28] that support this common pathophysiologic hypothesis.

**Table 3:** Common factors in the pathophysiology of RA and RA-ILD RA-ILD: Rheumatoid arthritis-associated interstitial lung disease Anti-CCP: Anti-cyclic citrullinated peptide [24]

Factors that are common in the pathophysiology of RA-ILD and RA
Identical citrullinated proteins are shared in both synovial and bronchial tissues in patients with RA.
In the pre-clinical phase of RA, the presence of anti-CCPs is associated with both airway disease and ILD.
Both RA factors and anti-CCP antibodies are found to be positive in lung tissues prior to being found positive in serum.
In patients with early RA, there is a local production of anti-CCP antibodies.
In patients with RA-associated ILD, a broader epitope has been found to be spreading.

In a contrasting study done in Japan [29], it was found that SE was associated with a reduced risk of RA-associated ILD. Other studies found that in the Japanese population, HLA-DRB 1*15 and *16 and HLA-DR2 were associated with a higher risk of developing RA-associated ILD [29,30] and UIP [31]. In the UK population, it was found that the risk of ILD in RA patients was higher with HLA-DRB1*07 [32]. Other mutations that are shared with RA-ILD are PARN, RTEL1, and TERT, as per a whole exome sequencing study [33].

Treatment of RA-ILD

Due to the paucity of randomized placebo-controlled trials, the management of RA-associated ILD is mostly empirical. In a patient presenting with an acute exacerbation of RA-ILD, high-dose corticosteroid therapy (1 mg/kg body weight of prednisolone or equivalent dose of another corticosteroid), combination therapy with immunosuppressive drugs such as mycophenolate mofetil (MMF), azathioprine, and cyclophosphamide (CYC), may also be administered in selected patients [34].

In patients with RA-associated ILD who received MMF therapy, a reduction in the corticosteroid dosage along with a substantial improvement in the diffusion capacity of carbon monoxide [DLCO] and forced vital capacity [FVC] was noted [34]. Two studies were done to assess the effect of rituximab in refractory RA-ILD. One was a study done at a single center that described no appreciable effects [35] on the disease course, while another case series showed moderate success post-therapy with rituximab [36]. Many studies are currently ongoing to assess the role of pirfenidone in RA-ILD. It is FDA approved for IPF, working as an anti-fibrotic drug through currently poorly understood pathways. An adequate response to immunosuppressants and steroids is seen in subjects with the non-UIP pattern. The factor of utmost importance in virtually all patients with RA-ILD is an urgent cessation of smoking.

Airway Diseases Associated with Rheumatoid Arthritis

RA can affect both the upper and lower respiratory tract, with the commonest mode of upper airway presentation being cricoarytenoid arthritis, which was found in nine out of 15 patients on HRCT of the neck, but only two patients were diagnosed on laryngoscopy examination, which might suggest that it is rare to find clinically significant involvement [37]. On HRCT examination, 39% to 60% of RA patients showed some evidence of airway involvement [38,39]. The clinical presentations of these patients can be dysphonia, dysphagia, odynophagia, or hoarseness of voice. In very rare cases, there can be significant upper airway obstruction, leading to airway compromise and the need for urgent endotracheal intubation. As per existing literature [40, 41], bronchiectasis and bronchiolitis are the primary manifestations of RA in the lower respiratory tract. In patients with RA, the prevalence of bronchiectasis leading to clinical manifestations has been shown to be 2.7%, as compared to a prevalence of 0.03% in the general population [42]. Some studies have reported findings of bronchiectasis long before clinically evident RA, while others have demonstrated bronchiectasis to be a late complication of RA [43,44]. Positive auto-antibodies and raised disease activity were found to have a higher association with bronchiectasis in patients with RA than in healthy controls [45]. Patients with RA treated with disease-modifying anti-rheumatic drugs (DMARDs) with underlying bronchiectasis are at a higher risk of infectious complications such as pneumonia. Mortality rates of more than 7.3 times as compared to the general population have been demonstrated in patients with RA with radiological findings of bronchiectasis [46]. In genetically predisposed subjects, chronic antigenic stimulation due to long-standing bacterial infection in bronchiectasis can lead to RA through immune-mediated pathways [47]. Life-long non-smoker patients with RA were found to have a higher prevalence of bronchiectasis at about 25% [48]. In patients with RA, airway pathology was found to be linked to SE [49], and this common genetic factor conferring increased risk can explain the association between RA and bronchiectasis. Small airway disease can manifest in patients with RA as obliterative or follicular bronchiolitis. Hyperplastic lymphoid cells with reactive germ cell centers within the bronchiolar walls are a characteristic histopathological finding in the follicular type of bronchiolitis [50]. The prognosis for both of these conditions has been reported to be dismal [51].

Vascular Diseases in Rheumatoid Arthritis

In patients with RA, the small to medium-sized blood vessels are primarily affected by destructive inflammatory infiltration. These changes are described pathologically as rheumatoid vasculitis. Cutaneous manifestations of vasculitis and neuropathy are more common as compared to primary pulmonary vasculitis. In patients with RA, findings of pulmonary arterial hypertension (PAH) are rarely seen.

Pulmonary manifestations in myositis

The predominant pulmonary manifestation in myositis has been found to be ILD. PAH may also be encountered in these patients but is generally a sequela of ILD [52]. The respiratory and pharyngeal muscles may be affected in myositis, leading to two crucial and serious complications: hypoventilation-associated respiratory failure and pneumonia secondary to aspiration.

Clinical and Epidemiological Characteristics of ILD Associated with Myositis

In patients with myositis, ILD prevalence ranges from 23% to 65% [53,54] but has been found to be as high as 70% when anti-aminoacyl tRNA synthetase antibody syndrome is present [55]. ILD is usually found initially in combination with early myositis but can manifest prior to or after musculoskeletal and dermatological manifestations are evident [53-57]. ILD associated with myositis usually presents with three characteristic patterns based on respiratory symptoms, namely: an asymptomatic form usually with subclinical disease; chronic disease with slow symptom progression; and rapidly progressive disease with an acute presentation [57]. The most common variant is the chronic form, which presents with a dry cough and gradually progressive dyspnea of insidious onset. Asymptomatic or sub-clinical ILD is present in about 30% of patients with dermatomyositis and polymyositis [58], and this relative absence of overt symptoms stresses the need for respiratory screening for all patients with myositis, particularly anti-Jo-1 antibody-positive patients. The histopathological finding in the rapidly progressive variant is diffuse alveolar damage [DAD], and this variant is usually accompanied by constitutional symptoms such as anorexia, lethargy, malaise, and fever [53]. ILD with a rapidly progressive course is well documented in patients with amyopathic dermatomyositis who present with the typical cutaneous manifestations of dermatomyositis such as a heliotrope rash and Gottron’s papules but have the absence of musculoskeletal symptoms [58,59]. These patients have an elevated mortality rate and an overall dismal prognosis with poor response in spite of aggressive therapy [53]. In all three clinical presentations, the five-year survival rates are about 70% [53]. In myositis-associated ILD, DAD and cryptogenic organizing pneumonia [COP] and DAD are frequently observed [60] in histologic patterns, in addition to UIP and NSIP [55]. Rapidly progressive ILD is also associated with anti-melanoma differentiation-associated gene 5 antibodies [anti-MDA5] [61,62].

Treatment of Myositis-Associated ILD

The first-line therapy in the management of myositis with ILD is high-dose corticosteroid therapy. When given monotherapy with corticosteroids, a response rate of about 50% has been demonstrated [57]. Augmentation therapy with immunosuppressive drugs such as MMF and cyclophosphamide has also been tried in myositis-ILD. Retrospective studies have shown the efficacy of rituximab in ILD patients [63]. Other biological therapies under evaluation for their effectiveness in myositis are abatacept, sifalimumab, and tocilizumab [64].

Pulmonary manifestations in systemic sclerosis (SSc)

Systemic sclerosis is an auto-immune disorder that is characterized by vascular injury, activation of immunological pathways, and fibrosis. The hallmark of the disease is fibrotic tissue changes predominantly in the skin, which may affect other internal organs. The main pulmonary manifestations of SSc are PAH and ILD, both of which contribute to increased mortality rates of 28% and 33%, respectively [65]. There are two subsets of SSc, namely, limited, which is associated with anti-centromere antibodies, and diffuse, which is associated with anti-topoisomerase I antibodies, both of which are associated with PAH and ILD. However, the limited subset shows a higher prevalence of PAH, while the diffuse subset shows a higher prevalence of ILD.

Systemic Sclerosis-Associated ILD (SSc-ILD)

The prevalence of ILD has been found to be 90% in SSc patients, although results vary on the basis of the imaging modality used and the demographic characteristics of the study population [66]. As compared to lung function tests, which may demonstrate high false negative rates in early stages, HRCT thorax has excellent sensitivity and specificity for the diagnosis of ILD, even in early disease stages [67]. On the basis of chest radiography alone, ILD was demonstrated in 35% of limited subset SSc patients in comparison to 53% in the diffuse subset, as per the European Scleroderma Trials and Research (EUSTAR) group [68]. Moderate to severe restrictive lung disease was seen in about 40% of SSc patients [69]. The majority of the decline in lung function is seen within the initial four to five years post-onset of non-Raynaud’s clinical features, after which the decline may show an indolent course [70]. The best predictors of the risk of disease progression have been found to be extensive pulmonary fibrosis and reduced forced vital capacity (FVC) values [71,72]. The various other risk factors conferring a higher risk of disease progression were identified to be male sex, abnormal capillaroscopy pattern of the nail folds, positive anti-topoisomerase antibodies (ATA), tobacco smoking, and African ethnicity [73,74]. Multiple cross-sectional studies have demonstrated that a greater magnitude of lung function impairment is seen in patients with findings of esophageal dilation on HRCT thorax [75,76]. On pulmonary function tests (PFT), esophageal dilation was shown to have a negative correlation with DLCO and FVC readings [75]. The characteristic findings on HRCT chest in SSc-associated ILD were found to be bronchogenic lung involvement with fibrosis in a centrilobular distribution [77].

These findings, correlating the esophageal diameter and degree of lung function impairment, can provide a plausible theory of micro-aspiration leading to disease progression in SSc-associated ILD. Both gastro-esophageal reflux disease (GERD) and micro-aspiration have been implicated in the pathogenesis of IPF [78]. The commonest disease pattern in SSc-associated ILD on both HRCT and histopathology has been found to be NSIP [66], and GGOs may also be seen, and the reversal is rarely seen even after therapy [79]. A systematic review and meta-analysis for evaluating the mortality in patients of SSc demonstrated that as compared to the general population, SSc was associated with higher mortality and that 74.9% of patients survived after five years from the time of diagnosis, as compared to 62.5% patients surviving after 10 years [80].

Genetic Mechanisms Involved in SSc-Associated ILD

Large-scale genetic studies have identified major histocompatibility complex (MHC) class II as the most important susceptibility locus in patients with SSc [81]. Other genes conferring susceptibility to SSc are also implicated in increased susceptibility to other auto-immune diseases such as multiple sclerosis (MS), primary biliary cirrhosis (PBC), RA, SLE, and inflammatory bowel disease (IBD) [81]. The susceptibility genes can be classified into two types based on their functions: the first being the genes involved in inflammatory and immunological mechanisms such as apoptosis, autophagy, and clearance of DNA (PPARG, FAS, RHOB, and ATG5); and the second, being those genes that promote fibrosis and extracellular matrix (ECM) deposition. Even though there is marked fibrosis in SSc, the genes involved in immunological mechanisms are far more as compared to fibrosis genes. Rare variants involved in coding for fibrosis can be detected by whole exome sequencing, which identifies the susceptibility genes involved in ECM-mediated pathways (COL5A2, COL22A1, COL13A1, COL4A4)[82]. In patients with SSc, various studies have shown that epigenetic mechanisms such as modification of histones, DNA methylation, and non-coding RNAs are also involved in fibroblastic cells and immune cells [83,84].

Management of SSc-Associated ILD

The focus of the physician should be to treat patients with significant respiratory symptoms who have extensive or progressive disease, as patients who have an FVC of more than 80% on initial evaluation seldom show a fall in pulmonary function [85]. The main therapeutic effect of immunosuppressive and anti-inflammatory therapy in SSc-associated ILD is to stabilize pulmonary function rather than target improvements in the same. In patients with symptomatic ILD, cyclophosphamide showed some effectiveness, with a difference of 2.5% at the end of one year in the mean of the absolute FVC values as compared to the placebo group, as per the Scleroderma lung study [86]. Based on two randomized controlled trials [RCT] demonstrating the superior efficacy of cyclophosphamide over placebo in SSc-associated ILD, the European League Against Rheumatism (EULAR) 2017 guidelines recommend cyclophosphamide therapy over MMF in patients with SSc-ILD [86,87]. In patients with comorbid conditions and a history of drug toxicities, first-line therapy with MMF can be initiated. Clinical trials are underway to investigate the role of biological therapy with rituximab and tocilizumab, and early reports are promising [88,89]. Combination therapy with MMF, pirfenidone, and nintedanib needs further studies in the treatment of SSc-associated ILD.

PAH Associated with Systemic Sclerosis

The hallmarks of PAH are vasoconstriction, in-situ thrombosis, and aberrant proliferation in the pulmonary arteries. A diagnosis of PAH requires that the mean pulmonary artery pressure (mPAP) be greater than 20 mm Hg on right heart catheterization (RHC), as per the updated hemodynamic definitions [90]. Isolated PAH is the commonest form of PAH that is encountered in SSc and is considered to be a major complication associated with higher mortality rates if not diagnosed and treated early. When patients with SSc were evaluated by RHC, the prevalence of PAH ranged from 8% to 12% in patients classified as high-risk on echocardiography or with low DLCO values on lung function testing [91,92]. However, the prevalence varies across different studies due to differences in diagnostic modalities used and population demographics. The limited variant of SSc has been shown to have a higher prevalence of PAH and has been found to be present in almost 50% of patients with CREST syndrome [93]. The mean time interval between the diagnosis of SSc and the development of clinically significant PAH has been shown to be 6.3 years [94] and is therefore considered a late complication of SSc. In SSc patients who develop PAH, the prognosis has been shown to be dismal, and the main factor contributing to this elevated death rate has been shown to be a delay in diagnosis, as symptoms are mainly seen in patients with advanced disease. The factors conferring an elevated risk of developing PAH in SSc patients [95] are summarized in Table 4.

**Table 4:** Risk factors for PAH associated with systemic sclerosis CREST: calcinosis, Raynaud's phenomenon, esophageal dysmotility, sclerodactyly, and telangiectasia SSc: systemic sclerosis [95]

Elderly patients
Male sex
Positive anti-centromere antibodies [ACA]
Positive anti-fibrillarin autoantibodies [AFA]
Digital ulcerations
CREST syndrome / Limited variant of SSc
Clinical findings of telangiectasia

Other laboratory tests that are highly suggestive of PAH are an out-of-proportion reduction in DLCO values and a rise in levels of N-terminal pro-brain natriuretic peptide (NT-pro BNP) levels [96]. An echocardiogram can be used as an initial screening tool for PAH by measurement of the velocity of the tricuspid regurgitation jet (TR), and a value of TR jet velocity of more than 2.5 m/sec is considered to be a diagnostic threshold for PAH. The sensitivity of echocardiography for diagnosing PAH ranges from 40 to 90% [97].

Treatment of SSc-PAH

As per the American College of Rheumatology (ACR) 2013 recommendations regarding clinical monitoring and screening for PAH in rheumatological disorders, screening evaluation should be done with echocardiography, NT-pro BNP, DLCO, and PFT in all systemic sclerosis patients who have clinical features such as abnormalities in nail fold capillaries, sclerodactyly, presyncope, dyspnea on exertion, and fatigue, as well as those patients with positive auto-antibodies specific to SSc [98]. In SSc-associated ILD, the first line of therapy is calcium-channel blockers (CCB), which have a vasodilatory action on the pulmonary vasculature. In the case of rapidly progressive disease, CCBs may prove to be insufficient in controlling the deranged hemodynamics due to PAH as well as in the resolution of clinical symptoms. Phosphodiesterase-5 inhibitors (PDE-5), endothelin-1 receptor antagonists (ETRA), and systemic therapy with prostacyclin analogs have also been used in treating PAH [99]. However, other than intravenous epoprostenol, no other therapy has shown any significant survival benefits in PAH [100]. As per the SERAPHIN (Symptomatic Pulmonary Arterial Hypertension) trial, Macitentan, which is a non-selective ETRA, was shown to have efficacy in reducing the risk of PAH-associated death, with a risk reduction of 30% with a dose of 3mg and almost 50% with a dose of 10mg [101]. Further research is needed to explore the role of combination therapy in treating PAH, which targets all three components of the pathogenesis of the disease, namely: autoimmune-mediated damage, fibrotic changes, and vascular injury.

Pulmonary manifestations of systemic lupus erythematosus (SLE)

As seen in other rheumatic disorders, estimating the extent of pulmonary involvement in SLE also depends on the diagnostic modality used and the population demographics. The most important diagnostic investigations are PFTs and HRCT thorax, which almost always reveal some pulmonary abnormalities even in an asymptomatic patient [102]. As seen in RA, the lung manifestations encountered in SLE can affect all the lung structure components: the airways, parenchyma, vasculature, and pleura [102].

Airway Diseases in SLE

Airway involvement is a relatively uncommon finding in SLE patients. However, when it is present, it usually involves both the upper and lower respiratory tracts. Involvement of the upper airways can lead to paralysis of the vocal cords, ulcerative lesions, and cricoarytenoid arthritis, and in rare cases, can cause necrotizing vasculitis with obstruction of the airways [103]. Coexistent bronchiectasis in patients with SLE can also be seen in imaging studies, although the clinical relevance of this finding is yet to be established [102]. Other rare airway presentations can include cryptogenic organizing pneumonia (COP) and bronchiolitis obliterans [104].

Lung Parenchymal Diseases in SLE

As compared to other connective tissue diseases, SLE patients have a lower prevalence of clinically significant ILD of up to 8% [105]. The most common ILD pattern has been found to be NSIP, while UIP has been found to be relatively uncommon [2]. In patients with progressive ILD with prominent clinical features, the management consists of immunosuppressive therapy with MMF, azathioprine, and cyclophosphamide, along with high-dose systemic corticosteroids.

Vascular Diseases in SLE

The primary manifestations of SLE in the lungs are diffuse alveolar hemorrhage (DAH) and PAH. The prevalence of PAH in patients with SLE has been demonstrated to be about 4% to 5% [106]. As per The Registry to Evaluate Early and Long-term PAH Disease Management (REVEAL registry), patients suffering from SLE with PAH had a one-year survival of about 94%, which was much better as compared to 82% in PAH associated with SSc [107]. A combination of vasodilatory drugs and immunosuppressive agents has been shown to be efficacious in the treatment of PAH in SLE patients [108]. DAH is considered to be a very rare but life-threatening manifestation of pulmonary involvement in SLE, with its estimated prevalence found to be less than 2% [109]. The mortality associated with DAH can be upwards of 50% [110]. The treatment in patients with DAH consists of high-dose systemic corticosteroids with immunosuppressive drugs along with intravenous immunoglobulins (IVIG) and plasma exchange, with studies showing a good response to rituximab therapy [111].

Pleural Diseases Associated With SLE

The commonest pleural pathology, found in almost half of all SLE patients, is pleural effusion [112]. Effusions can be both unilateral as well as bilateral. Laboratory examination of the pleural fluid in SLE-associated pleuritis is essential and usually reveals an exudative fluid with a raised total leucocyte count, predominantly lymphocytes or neutrophils, and a glucose level that can be either low or normal [113]. Further pleural fluid diagnostic testing for anti-nuclear antibodies (ANA) should be undertaken to narrow down the differential diagnosis, and an elevated ANA titer (greater than 1:160) is strongly suggestive of lupus pleuritis [114]. Pleural effusions that range from moderate to severe are usually responsive to oral corticosteroid therapy, while mild effusions show a good response to non-steroidal anti-inflammatory drugs (NSAIDs).

A rare and characteristic manifestation found in SLE patients is known as "shrinking lung syndrome". It is characterized clinically by shortness of breath, progressive reduction in lung volumes identified as an elevation of the diaphragm on chest x-ray or a restrictive pattern on PFT, and pleuritic chest pain. However, shrinking lung syndrome is a rare entity in SLE patients and is encountered in around 1% of SLE patients [115]. The various hypotheses for the pathogenesis of shrinking lung syndrome in SLE are summarized in Table 5.

**Table 5:** Possible mechanisms involved in the pathogenesis of shrinking lung syndrome in SLE [115]

Weakness of the respiratory muscles
Phrenic nerve palsy
Fibrotic changes in the diaphragm
Micro-atelectasis due to surfactant reduction
Inflammation of the pleura

Treatment of shrinking lung syndrome involves the use of high-dose corticosteroid therapy, which has been demonstrated to have fairly good success rates [116]. Augmentation therapy with immunosuppressants may be considered in refractory cases, particularly rituximab [116]. Other strategies to improve diaphragmatic dysfunction are chest physiotherapy and the use of beta-adrenergic receptor agonists and theophylline.

## Conclusions

The four main rheumatological disorders that affect the lungs are rheumatoid arthritis, systemic lupus erythematosus, myositis, and systemic sclerosis. Of these diseases, each demonstrates a characteristic pattern of pulmonary involvement and affects different structural components of the lung. The incidence, prevalence, response to treatment, and disease severity also vary in all these four diseases concerning the lung structures affected. The most common pulmonary involvements that are associated with increased morbidity and mortality are PAH and ILD. Early screening for the same with HRCT, PFT, and DLCO in patients with rheumatological diseases can enable early diagnosis and rapid treatment initiation, which can improve the prognosis for the patient and slow down the disease progression. A multi-disciplinary approach with a team consisting of a pulmonologist, rheumatologist, radiologist, and cardiologist is essential to tackle the various clinical manifestations of these auto-immune diseases. A good understanding of the underlying disease epidemiology and genetics can lead to the development of specific gene-targeted therapies in the future.
